# The comparative effectiveness of Integrated treatment for Substance abuse and Partner violence (I-StoP) and substance abuse treatment alone: a randomized controlled trial

**DOI:** 10.1186/1471-244X-13-189

**Published:** 2013-07-16

**Authors:** Fleur L Kraanen, Ellen Vedel, Agnes Scholing, Paul MG Emmelkamp

**Affiliations:** 1 Department of Clinical Psychology, University of Amsterdam, Amsterdam, The Netherlands; 2 Cognitive Science Center Amsterdam, University of Amsterdam, Amsterdam, The Netherlands; 3 Forensic Outpatient Clinic De Waag, Amsterdam, The Netherlands; 4 Jellinek Substance Abuse Treatment Centre, Arkin, Amsterdam, The Netherlands; 5 King Abdulaziz University, Jeddah, Saudi Arabia

**Keywords:** Substance use disorders, Intimate partner violence, Perpetration, Treatment, Randomized controlled trial, Comorbidity, Cognitive behavior therapy

## Abstract

**Background:**

Research has shown that treatments that solely addressed intimate partner violence (IPV) perpetration were not very effective in reducing IPV, possibly due to neglecting individual differences between IPV perpetrators. A large proportion of IPV perpetrators is diagnosed with co-occurring substance use disorders and it has been demonstrated that successful treatment of alcohol dependence among alcohol dependent IPV perpetrators also led to less IPV. The current study investigated the relative effectiveness of Integrated treatment for Substance abuse and Partner violence (I-StoP) to cognitive behavioral treatment addressing substance use disorders including only one session addressing partner violence (CBT-SUD+) among patients in substance abuse treatment who repeatedly committed IPV. Substance use and IPV perpetration were primary outcome measures.

**Method:**

Patients who entered substance abuse treatment were screened for IPV. Patients who disclosed at least 7 acts of physical IPV in the past year (N = 52) were randomly assigned to either I-StoP or CBT-SUD+. Patients in both conditions received 16 treatment sessions. Substance use and IPV perpetration were assessed at pretreatment, halfway treatment and posttreatment in blocks of 8 weeks. Both completers and intention-to-treat (ITT) analyses were performed.

**Results:**

Patients (completers and ITT) in both conditions significantly improved regarding substance use and IPV perpetration at posttreatment compared with pretreatment. There were no differences in outcome between conditions. Completers in both conditions almost fully abstained from IPV in 8 weeks before the end of treatment.

**Conclusions:**

Both I-StoP and CBT-SUD+ were effective in reducing substance use and IPV perpetration among patients in substance abuse treatment who repeatedly committed IPV and self-disclosed IPV perpetration. Since it is more cost and time-effective to implement CBT-SUD+ than I-StoP, it is suggested to treat IPV perpetrators in substance abuse treatment with CBT-SUD+.

**Trial registration:**

ClinicalTrials.gov: NCT00847873

## Background

Intimate partner violence (IPV) perpetration is highly prevalent among substance abusers. Several studies demonstrated that up to more than half of male and female patients in substance abuse treatments committed at least one act of physical IPV in the past year (e.g., [1-5]; Kraanen, Vedel, Scholing, Emmelkamp: Do specific substance use disorders or combinations of specific substance use disorders predict past year intimate partner violence in patients referred to substance abuse treatment?, submitted). These figures are substantially higher than in the normal population, where about 20% of the population committed IPV in the past year [6]. Also, substance abuse is overrepresented in male and female IPV perpetrators in batterers’ treatment [7-13]. The relationship between substance use (particularly alcohol use) and IPV received increased attention over the past years. Several reviews confirmed the association between alcohol use and IPV (e.g., [14-16]). In addition, a number of studies demonstrated that IPV perpetration in alcohol dependent patients decreased substantially after successful treatment for substance abuse (for reviews, see: [17,18]). These results were found when substance abusers were treated individually [4,19] as well as when participants had received couples therapy [20-22]. Moreover, patients who relapsed to alcohol use after successful alcohol use disorder treatment were much more likely to relapse to IPV perpetration than patients who abstained from alcohol [23,24].

Also, the use of illicit drugs (particularly cocaine and cannabis) is connected to IPV perpetration (for reviews, see: [15,25-27]). Presumably, different routes lead from cocaine use and cannabis use to IPV perpetration [25]. For cocaine it is hypothesized that its psychopharmacological effects *directly* increase aggressive behavior (e.g., [28,29]), whereas for cannabis it is assumed that irritability as a result of *withdrawal* may lead to committing IPV [30]. To our knowledge, it has not been studied yet whether successful treatment of cannabis and cocaine use disorders in patients who are referred for substance use treatment and commit IPV, also reduces IPV perpetration.

Results described above indicate that successful treatment of alcohol dependence in substance abusing IPV perpetrators leads to reduced IPV perpetration. Particularly since there are no evidence-based treatments for IPV perpetrators, these results are valuable. Several meta-analyses examined the effectiveness of IPV treatment; feminist psychoeducation (Duluth model) as well as cognitive behavioral interventions were found to have effect-sizes near zero [31,32] and were thus hardly effective in reducing IPV. There are some indications that couples therapy aiming to reduce IPV may be promising for specific couples and types of IPV [33,34], but it is too early to draw firm conclusions. One major problem of the early treatment studies on IPV is that most treatments were offered in a group format, followed a one-size-fits-all approach, and did not take individual characteristics of patients (such as substance use) into account. Recently, several authors (e.g., [35-37]) claimed that treatments for IPV should be more effectively tailored to specific patient and couple characteristics.

Because treatment for alcohol use disorders is effective in reducing IPV perpetration in alcohol abusing patients referred for substance use treatment whereas IPV treatment alone is not, several researchers called for integrating IPV and substance abuse treatment (e.g., [38-41]). However, to date, only one randomized controlled trial (RCT) has been carried out on the effectiveness of such an integrated treatment. [42] conducted a pilot study that compared the effectiveness of a combined alcohol dependence / domestic violence group therapy based on cognitive behavioral therapy (CBT) to a 12-step facilitation group that did not address partner violence. Participants who received the combined treatment abstained significantly more days from alcohol than participants in the 12-step facilitation group and there was a trend for participants in the combined treatment to engage in less frequent IPV than participants in the 12-step group. However, since there were differences in days of abstinence between both conditions and alcohol use is possibly causally related to IPV perpetration, it is necessary to control for days of abstinence when assessing differences between treatments in IPV perpetration. Further, in [42]’s study the treatments for substance abuse in both conditions differed from one another (i.e., CBT vs. 12-step approach). Therefore, it was not possible to determine whether reductions in IPV were attributable to the different treatment of substance abuse or to the focus on IPV in the combined treatment. Also, some participants had no actual intimate relationship. Although these participants may benefit from the treatment in future relationships, it is not possible to measure reductions in IPV perpetration when no partner is present. In addition, only participants who were (also) diagnosed with alcohol dependence were included, whereas research has demonstrated a relationship between use of cocaine and cannabis and IPV perpetration as well. Besides, only men were included whereas a substantial proportion of the IPV perpetrators consist of women [43,44].

The current study compared CBT addressing both substance use disorders and IPV (Integrated treatment for Substance abuse and Partner violence; I-STOP; [45,46]) to CBT addressing only substance use disorders plus one session addressing IPV for enhancing safety (CBT-SUD+). The interventions addressing substance use were equal in both conditions; CBT-SUD+ contained the same topics as I-StoP regarding substance use but had more sessions to address these topics. Further, only participants who actually were in an intimate relationship with the victim at the start of treatment were included. In addition, participants with other (primary) substance use disorders than alcohol dependence could participate. The study was open for male and female patients, and treatments were conducted individually.

Aim of this study was to compare the effectiveness of IPV perpetration between I-StoP and CBT-SUD+ on IPV perpetration and reduction of substance abuse among patients in substance abuse treatment who were involved in a pattern of IPV perpetration. It was hypothesized that 1) participants’ substance use in both conditions would be significantly reduced at posttreatment compared to pretreatment, 2) there would be no significant differences between conditions regarding substance use at posttreatment, 3) physical IPV perpetration would be significantly reduced at posttreatment compared to pretreatment in participants in both conditions, and 4) patients receiving I-StoP would engage in less frequent IPV at posttreatment compared to patients allocated to CBT-SUD+. Effects on secondary outcome measures (verbal IPV, inflicted injuries, general mental health, marital satisfaction, and treatment satisfaction) were studied exploratory.

## Methods

### Participants

Participants were recruited from patients who sought treatment at a substance abuse treatment facility (Jellinek) in Amsterdam, the Netherlands, between August 1, 2009 and June 1, 2012. Patients were included if they 1) disclosed 7 or more acts of physical IPV in the past year at intake, 2) were diagnosed with abuse and / or dependence of alcohol, cannabis and / or cocaine, 3) were in an intimate relationship with the partner against whom they committed IPV, and 4) were triaged to outpatient treatment. We aimed to include patients who committed at least 7 acts of IPV in the past year since the goal of the treatment was to break a *pattern* of IPV perpetration in an enduring relationship. Therefore, we intended not to include patients to whom IPV perpetration was an incident. Patients who were involved in incidental IPV typically reported 2 or 3 acts of physical IPV (for example, they reported to have pushed, grabbed, and slapped their partner). To select patients who were involved of a pattern of IPV perpetration we decided to select a cutoff that was well above 3 and settled for a cutoff of 7. In addition, patients were recruited on the basis of *past year* IPV, but IPV as treatment outcome was assessed in blocks of 8 weeks. Another reason to include only patients who were involved in a pattern of IPV perpetration was to minimize the chance that patients would report no acts of physical IPV at pretreatment and could thus not improve during treatment. Finally, only patients who were able to follow outpatient treatment were invited to participate because inpatients were offered various other treatment modules besides substance abuse treatment, including social skills training and emotion regulation treatment, which would overlap with I-StoP. Patients were excluded if they 1) were diagnosed with crack cocaine or heroin abuse or dependence, 2) *currently* received treatment for other mental health problems, 3) had insufficient knowledge of the Dutch language to complete questionnaires, and 4) in case of severe mental health problems (e.g., psychosis, suicidal ideation) or cognitive disorders (e.g., Korsakoff’s syndrome). Patients were excluded in case of crack cocaine and/or heroin use disorders because these patients usually need more intensive treatment than outpatient treatment. Initially it was aimed to include 100 participants in the current study.

### Treatments

Two treatment protocols were developed for this study, i.e., I-StoP and CBT-SUD+. Both treatments consisted of 16 sessions of 45 minutes and were ideally delivered weekly to the participants. Treatments were conducted individually, but the partner was invited to attend the first session of both I-StoP and CBT-SUD+ in order to check and enhance safety. I-StoP and CBT-SUD+ were flexible treatment protocols, i.e., treatment could be adjusted according to motivation and / or level of functioning of the patient. Furthermore, sessions could be modified in case of crisis, such as relapse to substance use or IPV, or topics could be treated in different order if relevant.

#### I-STOP

I-STOP concurrently addressed both substance abuse and IPV perpetration. Substance abuse interventions were based on evidence-based CBT protocols addressing substance abuse adapted for use in The Netherlands [47-50]; for a description of interventions, see [51]). Interventions targeting IPV comprised CBT and were based on the work of Dutton [52,53]. Motivational interviewing techniques [54] were included to increase participant’s motivation to change substance abuse and stop IPV perpetration. Sessions primarily targeting IPV and substance use were alternated. In addition, participants received a workbook containing psychoeducation, weekly assignments that correspond to the central topic of a session, and diary cards to daily register substance abuse / craving *and* anger / perpetration of IPV. Therapists were instructed to address both IPV and substance abuse in each session by emphasizing anger / IPV registrations if the central theme of a session was related to substance abuse, and vice versa.

Each session followed the same structure: 1) addressing completed diary cards and assignments, 2) discussing the main topic of the session, and 3) explaining the assignment for the next session. I-StoP contained the following interventions. First, the ‘Cycle of Violence’ [55] was discussed with the patient and partner and the couple was taught to take time-outs in case of high-risk situations for IPV. It was important that the partner was present in order to maximize the chance that the time out was going to be successful and to minimize the risk that the patient would use the tool abusively, for example, to control the interaction with the partner (for a more detailed description of the negotiated time-out procedure, see [56]). In addition, the following topics and interventions were addressed: explanation of the treatment rationale, assessment of types of IPV that took place in the relationship, assessment of pros and cons of IPV and substance abuse, formulating treatment goals regarding IPV (abstaining from IPV was the only adequate treatment goal) and substance use (preferable abstinence, but controlled substance use was also an accepted treatment goal; see [51]), identifying self-control measures to prevent substance use, making functional analyses of substance use, anger management, coping with craving and emotions that may lead to substance use, the association between thoughts, feelings, and behavior in relation to substance use and IPV, communication skills, and relapse prevention for IPV and substance use.

#### CBT-SUD+

CBT-SUD+ is a manualized, cognitive behavioral treatment that can be considered treatment-as-usual for substance use treatment in The Netherlands. For ethical reasons (checking and promoting safety), the first session of CBT-SUD+ is the same as the first session of I-STOP (i.e., a session addressing the ‘Cycle of Violence’ and time-out procedure with the partner). Sessions followed the same structure as I-StoP-sessions. Further, topics and interventions of CBT-SUD+ were the same as I-STOP interventions addressing substance abuse. However, since CBT-SUD+ treatment consisted of 16 sessions as well, there was twice as much time to discuss these topics.

#### Therapists and treatment adherence

Five female social workers, who had received formal training in CBT and motivational interviewing and who had extensive experience in substance abuse counseling, were trained in both treatment protocols. PE and FK supervised therapists once every two weeks during supervision sessions lasting 90 minutes. All patients were discussed comprehensively during supervision and meetings focused on adherence to treatment manuals and preparation of future sessions.

#### Ethics and randomization

The study was registered at the clinical trials registry (http://www.clinicaltrials.gov) (# NCT00847873) and was ap-proved by the Ethics Review Board of the University of Amsterdam (2008-KP-466). Participants were randomly assigned to either I-STOP or CBT-SUD+ using http://www.randomization.com. Outcome of randomization was written on cards and put in a closed envelope containing participant numbers. Envelopes were handed to participants after completing pretreatment assessment.

### Measures

#### Outcome measures

##### IPV

The Revised Conflict Tactics Scales (CTS2; [57]) was used to assess frequency and prevalence of IPV. The CTS2 consists of 39 item pairs addressing both perpetration and victimization of a specific act of violence. An example is: ‘I pushed my partner’ and ‘My partner pushed me’. Answers are scored on a 7-point scale: 0 = never; 1 = once; 2 = twice; 3 = 3–5 times; 4 = 6–10 times; 5 = 11–20 times and 6 = more than 20 times. The CTS2 comprises of 5 scales measuring different aspects of handling conflicts between partners: 1) physical violence, 2) verbal violence, 3) sexual violence, 4) negotiation, and 5) injuries resulting from IPV. Frequency scores of violent acts were calculated by taking the average of the frequency range (e.g., 3–5 times = 4), as recommended by [57]. The CTS2 is worldwide the scale that is most often used to measure the frequency, type, and gravity of IPV. Previous research showed that the CTS2 is reliable and valid for this goal (e.g., [57-61]). As primary outcome measure, the physical violence subscale was used; the verbal violence and injuries scales were used to assess secondary treatment outcomes. The CTS2 was administered pretreatment, halfway treatment (after session 8), and posttreatment. The partner was invited to complete the CTS2 pretreatment and posttreatment as well. On every occasion IPV was assessed with reference to the past 8 weeks.

##### Substance use

To assess substance use, the Timeline Follow Back Interview (TLFB; [62]) and the Quick Drinking Screen (QDS; [63]) were used. The TLFB is a calendar-based method to assess frequency and quantity of substance use and days of abstinence [64]. The TLFB is often studied and most studies found the TLFB to be a highly valid method to assess alcohol (e.g., [65,66]) and drug use (for a review, see [67]). The instrument was administered pre- en posttreatment to assess substance use in 8 weeks prior to treatment and 8 weeks before the end of treatment. In addition, the QDS was administered. The QDS is a self-report instrument that contains 5 aggregate summary questions regarding alcohol use. Data obtained by the QDS were found to be very similar to data obtained by the TLFB [63,68,69]. The QDS was modified to assess drug use as well. Participants were asked about substance use in the past 8 weeks. The modified QDS was administered to the participant pretreatment, halfway treatment (after session 8), and posttreatment, and assessed substance use in the past 8 weeks.

##### General psychopathology

To assess general psychopathology, the Brief Symptom Inventory (BSI; [70]; Dutch translation: [71]) was used. The total BSI score provides an overall measure of severity of psychopathology. Psychometric qualities of the BSI are good [72]. Participants completed the BSI at pre- and posttreatment; the BSI assessed symptoms of psychopathology during the past week.

##### Marital satisfaction

The marital maladjustment-scale of the Dutch version of the Maudsley Marital Questionnaire (MMQ; [73]) was used to assess marital satisfaction. Psychometric qualities are good (e.g., [73,74]). Participants completed the MMQ at pre- and posttreatment.

#### Treatment satisfaction

Participants were asked at posttreatment to rate treatment satisfaction on a scale from one to ten.

### Clinical diagnoses and assessment of eligibility

#### Patient characteristics

The Measurements in the Addictions for Triage and Evaluation (MATE; [75]) was used to assess patient characteristics and to guide treatment allocation (i.e., allocation to inpatient or outpatient treatment).

#### IPV

The Jellinek Inventory for assessing Partner Violence (J-IPV; [76]) was used to screen patients for past year IPV perpetration. The J-IPV consists of 4 items; a positive answer to at least one item is indicative for IPV perpetration. The J-IPV possesses good psychometric properties to screen for IPV perpetration. In addition, the CTS2 (further named CTS2-screen to distinguish its purpose here from the CTS2 when used as outcome measure) [56] was administered to assess type and frequency of acts of past year IPV perpetration; patients had to have engaged in at least 7 acts of IPV in the past year to be included in the study.

#### Axis-I disorders

Axis-I disorders were classified using the Structured Clinical Interview for DSM-IV Axis-I Disorders (SCID-I; [77]; Dutch translation: [78]).

### Procedure

At the intake, the MATE was administered, followed by the J-IPV. Patients who answered positive to one or more J-IPV items were invited for a second intake, which involved administration of the SCID-I and CTS2-screen. Participants who met inclusion criteria and were willing to participate were scheduled for pretreatment assessment. During this meeting, first, the purpose of the study was explained and informed consent was obtained. Then, the TLFB was administered and patients consequently completed the BSI, the QDS, the CTS2, and the MMQ. After that, participants were handed an envelope containing the treatment condition they were randomly assigned to (either I-StoP or CBT-SUD+) and an appointment for participant and partner was scheduled with one of the therapists. After that, treatment started. After session 8, the therapist asked participants to complete the modified QDS and the CTS2. After session 16, an appointment was scheduled for posttreatment assessment, during which, consequently, the TLFB, the BSI, the QDS, the CTS2, and the MMQ were completed. There was no compensation for treatment participation or completion of assessments by patients who completed treatment; patients who dropped out from treatment received 15 euro (about 20 US dollar) if they completed posttreatment assessment.

### Statistical analyses

#### Participant characteristics

Demographics, current Axis-I disorder diagnoses, frequency of past year IPV and number of dropouts were compared between conditions using chi-square-tests for dichotomous variables, and t-tests and Mann–Whitney tests for normally and nonnormally distributed continuous variables, respectively.

#### Treatment effects

For the analysis of treatment effects, both completers and intention-to-treat (ITT) analyses were conducted. For ITT analyses, the last observation carried forward (LOCF) method was used. Patients were classified as completer if they attended at least 75% of treatment sessions. Patients were categorized as ITT if at least one treatment session was attended. Two measures were used to assess changes in substance use: days of abstinence and the average quantity of substances that were weekly used (since controlled substance use was also an accepted treatment goal). Since participants used different substances (measured on different scales, e.g. units or grams) and -in several cases- more than one substance, it was necessary to calculate new (standardized) Z-scores for substance use. First, pre- and posttreatment quantities of 1) alcohol (mean standard units per week), 2) cannabis (mean grams per week), and 3) cocaine (mean grams per week) that had been used by all patients in the preceding 8 weeks were each displayed in a separate column. Then, the data in each column were transformed to Z-scores, after which the Z-scores in each column were again split in the pre- and posttreatment scores. Finally, for pretreatment and posttreatment separately, the Z-scores for alcohol, cannabis, and cocaine were added up in order to obtain a quantity-frequency summary measure of the combined use of different substances at both pretreatment and posttreatment. Days of abstinence and Z-scores were calculated on the basis of the QDS (instead of the TLFB) because QDS scores obtained halfway treatment were of use for the LOCF procedure (the TLFB was administered only at pre- and posttreatment). TLFB outcomes were not further used. IPV outcomes at pretreatment, halfway treatment and posttreatment were assessed by calculating frequency scores of physical IPV (primary outcome measure), verbal IPV, and injuries inflicted to the partner within the preceding 8 weeks.

Pre- and posttreatment measures of substance use and IPV were compared within treatments using one-tailed paired samples t-tests for normally distributed values and one-tailed Wilcoxon signed rank tests for non-normally distributed values. ANCOVA’s using pretreatment substance use / IPV as covariate were carried out to assess between-treatment differences because initial analyses showed a correlation between the pre- and posttreatment scores on both substance use and IPV, respectively. For a few ANCOVA’s the assumption of normality was violated. However, since sample sizes were roughly equal it was assumed that the F-statistic would be relatively robust against violations of normality [79]. Further, Levene’s test was used to assess homogeneity of variance. Finally, homogeneity of regression slopes was assessed; for some analyses for the ITT sample this assumption was violated. However, ANCOVA results are relatively unaffected by violations of the assumption of homogeneity of regression when group sizes are equal [80-82]. Based on the above, there was no need to refrain from using ANCOVA’s.

#### Treatment effects–secondary outcome measures

Pre- and posttreatment BSI and MMQ scores to assess overall psychopathology and marital satisfaction, respectively, were compared within treatments using paired samples t-tests and Wilcoxon signed rank tests; ANCOVA’s with pretreatment outcomes as covariate were performed to assess differences in posttreatment BSI and MMQ scores between treatments. An independent samples Mann–Whitney test was used to compare treatment satisfaction across both treatments.

#### Power analyses

We argued that to be *clinically* meaningful, the difference between pre- and posttreatment IPV should be large. For example, a small decrease in IPV might be statistically significant, but if an IPV perpetrator abuses a partner 3 times instead of 4 times in 8 weeks we did not consider this of clinical importance. The same applied to the difference between treatments. If, for example, IPV would decrease only slightly more in the I-StoP condition than in the CBT-SUD+ condition, these results would not match the effort needed to implement I-StoP in routine clinical care. The number of participants that were needed for a power of .80 was determined using G*Power [83]. The analyses demonstrated that 12 participants per condition were necessary to detect a large difference within conditions (Cohen’s d > .80; [84]) at α = .05, one-sided testing; 21 participants per condition were needed to detect a large difference between conditions including one covariate (i.e., a difference between patients who received I-StoP and patients who received CBT-IPV+ after controlling for pretreatment values; Cohen’s f > .40; [84]) at α = .05, one-sided testing.

## Results

### Participant characteristics

**Figure 1:**
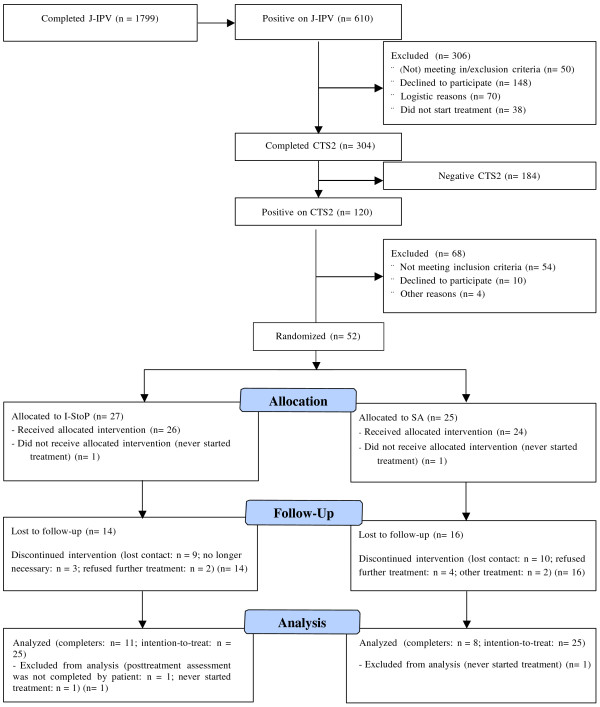
CONSORT 2010 flow chart of participant enrollment, randomization, and retention of the current study.

Figure 1 displays the CONSORT 2010 flow diagram of participants from IPV screening through treatment completion. The final sample consisted of 52 patients who were included in the treatment study; 27 and 25 participants were randomly assigned to I-StoP and CBT-SUD+ respectively. Table 1 displays demographics and clinical characteristics of participants. There were no statistically significant differences between participants receiving I-StoP and participants receiving CBT-SUD+.

**Table 1:** Demographics and clinical characteristics of patients at pretreatment

	**I-StoP**	**CBT-SUD+**	**Overall**	
	**(N = 27)**	**(N = 25)**	**(N = 52)**	**Between group analyses**
	**N (%)**	**N (%)**	**N (%)**	
Gender				
Male	19 (70.4)	17 (68.0)	36(69.2)	X^2^ (1) = 0.03; p = .85
Female	8 (29.6)	8 (32.0)	16 (30.8)	
Age (M, SD)	34.85 (9.87)	37.08 (8.87)	36.18 (9.29)	t (50) = 0.43; p = .51
Relationship length (years) (M, SD)	5.08 (4.59)	7.94 (8.59)	6.48 (6.93)	U = 270.00; p = .55
Current alcohol use disorder	22 (81.5)	19 (76.0)	41 (78.8)	X^2^ (1) = 0.23; p = .63
Current cannabis use disorder	13 (48.1)	8 (32.0)	21 (40.4)	X^2^ (1) = 1.41; p = .24
Current cocaine use disorder	6 (22.2)	9 (36.0)	15 (28.8)	X^2^ (1) = 2.02; p = .16
Number of SUDs (M, SD)	1.48 (0.64)	1.44 (0.58)	1.46 (0.61)	U = 330.50; p = .88
Current major depressive episode	4 (15.4)	6 (24.0)	10 (19.6)	X^2^ (1) = 0.23; p = .63
Current panic disorder	1 (3.8)	0 (0.0)	1 (2.0)	X^2^ (1) = 0.98; p = .32
Current social phobia	2 (7.7)	5 (20.0)	7 (13.7)	X^2^ (1) = 1.63; p = .20
Current OCD	0 (0.0)	2 (8.0)	2 (3.9)	X^2^ (1) = 2.17; p = .14
Current PTSD	1 (3.8)	4 (16.0)	5 (9.8)	X^2^ (1) = 2.13; p = .15
Current GAD	1 (3.8)	0 (0.0)	1 (2.0)	X^2^ (1) = 0.98; p = .32
Past year physical IPV perpetration	32.37 (47.62)	23.00 (23.20)	27.87 (37.84)	U = 334.50; p = .96
Past year verbal IPV perpetration	69.19 (43.58)	52.24 (27.54)	61.04 (37.39)	U = 2.96.00; p = .21
Past year sexual IPV perpetration (n = 51)	2.65 (9.90)	0.72 (2.51)	1.71 (7.28)	U = 301.50; p = .48
Past year inflicted injuries (n = 51)	3.50 (3.96)	4.72 (6.63)	4.10 (5.42)	U = 296.50; p = .59
Underwent clinical detoxification^1^	6 (24.0)	6 (25.0)	12 (24.5)	X^2^ (1) = 0.01; p = .94

^1^ = clinical detoxification took place during the course of treatment if it appeared that patients could not obtain their treatment goal at home and lasted 1 week; I-StoP = Integrated treatment for Substance abuse and Partner violence; CBT-SUD+ = substance abuse treatment; SUD = substance use disorder; OCD = obsessive compulsive disorder; PTSD = posttraumatic stress disorder; GAD = generalized anxiety disorder; IPV = Intimate partner violence.

### Retention

A total of 19 participants (36.5%) completed at least 75% of treatment sessions (I-StoP: N = 11 (40.7%); CBT-SUD+: N = 8 (32.0%)). Dropout rates did not differ significantly between I-StoP and CBT-SUD+ (p = .51). Two out of 52 participants (1 in each condition) completed the pretreatment assessment but never started treatment and were excluded from further analyses. Reasons for dropout and other information regarding treatment completion/dropout are displayed in Table 2. There were no significant differences between conditions.

**Table 2:** Reasons for dropout and completion rates per condition

	**I-StoP**	**CBT-SUD+**	**Overall**	
	**N = 25**	**N = 24**	**N = 49**	**Between group analyses**
	**N (%)**	**N (%)**	**N (%)**	
Reasons for drop-out				
Patients could not be contacted	9 (36.0)	10 (41.7)	19 (38.8)	X^2^ (4) = 6.18; p = .19
Agreement treatment was no longer needed	3 (12.0)	0 (0.0)	3 (6.1)	
Did not want treatment anymore	2 (8.0)	4 (16.7)	6 (12.2)	
Needed different treatment	0 (0.0)	2 (8.3)	2 (4.1)	
No. of sessions attended–ITT M (S.D.)	9.25 (6.54)	8.68 (5.59)	8.96 (6.02)	U = 293.50; p = .89
No. of sessions attended–completers M (S.D.)	16.00 (0.00)	15.50 (1.41)	15.79 (0.92)	U = 38.50; p = .68
No. of partners that attended first session	15 (60.0)	12 (50.0)	27 (55.1)	X^2^ (1) = 0.50; p = .48
Relationship intact at posttreatment (completers)	24 (96.0)	24 (100.0)	48 (98.0)	X^2^ (1) = .98; p = .32
Treatment duration–ITT (weeks) M (S.D.)	22.19 (14.43)	20.21 (12.44)	21.22 (13.39)	t (47) = 0.51; p = .61
Treatment duration–completers (weeks) M (S.D.)	32.31 (12.53)	33.93 (6.80)	32.99 (10.22)	U = 30.00; p = .25
Completed pretreatment assessment (t1)	25 (100.0)	24 (100%)	49 (100%)	-
Completed halfway treatment assessment (t2)	11 (44.0%)	13 (54.2%)	24 (49.0%)	X^2^ (1) = 0.51; p = .48
Completed posttreatment assessment (t3)	11 (44.0%)	8 (33.3%)	19 (38.8%)	X^2^ (1) = 0.59; p = .44

No. = number; I-StoP = Integrated treatment for Substance abuse and Partner violence; CBT-SUD+ = substance abuse treatment.

### Primary outcomes

#### Substance use

Days of abstinence and substance use Z-scores of completers and ITT sample are displayed in Table 3.

**Table 3:** Pre- and posttreatment substance use (QDS) per condition

	**Completers**		**Intention-to-treat**	
**Variable**	**I-StoP**	**CBT-SUD+**	**I-StoP**	**CBT-SUD+**
	**M (SD)**	**M (SD)**	**M (SD)**	**M (SD)**
Days abstinence 8 weeks pretreatment	20.36 (23.27)	17.50 (16.55)	16.48 (10.14)	11.67 (14.96)
Days abstinence 8 weeks posttreatment	35.27 (20.54)	40.00 (18.27)	25.28 (22.23)	23.50 (20.54)
Z-scores 8 weeks pretreatment	1.55 (3.81)	0.13 (1.06)	0.34 (2.18)	0.33 (1.57)
Z-scores 8 weeks posttreatment	−0.73 (1.19)	−1.19 (0.58)	−0.43 (1.45)	−0.26 (1.76)

I-StoP = Integrated treatment for Substance abuse and Partner violence; CBT-SUD+ = substance abuse treatment.

In both treatment conditions the completers had been significantly more days abstinent at posttreatment than at pretreatment (I-StoP: Z (10) = −1.99; p = .02 (1-tailed); SA: (t (7) = −4.17; p = .00). Comparable findings were found on substance use Z-scores at posttreatment versus pretreatment (I-StoP: Z (10) = −2.05; p = .02 (1-tailed); CBT-SUD+: Z (7) = −2.37; p = .01 (1-tailed)). There were no statistically significant differences between I-StoP and SA in posttreatment days of abstinence and substance use Z-scores after controlling for pretreatment values. ITT analyses demonstrated similar results. Participants receiving I-StoP (Z = −2.13; p = .02; 1-tailed) as well as CBT-SUD+ (Z = −3.08; p = .00; 1-tailed) had been significantly more days abstinent at posttreatment than at pretreatment. Also, participants allocated to I-StoP (Z = −1.97; p = .02; 1-tailed) as well as CBT-SUD+ (Z = −2.72; p = .00; 1-tailed) had significantly lower substance use Z-scores at posttreatment than at pretreatment. Again, there were no differences between both conditions in days of abstinence and substance use Z-scores after controlling for pretreatment values.

#### IPV

Table 4 displays physical and verbal IPV perpetration and injuries inflicted to the partner for both treatment groups, at pretreatment and posttreatment and for completers and ITT sample.

**Table 4:** IPV perpetration 8 weeks before pretreatment assessment and 8 weeks before posttreatment assessment of completers and ITT sample per condition

	**Completers**		**Intention-to-treat sample**	
	**I-StoP**	**CBT-SUD+**	**I-StoP**	**CBT-SUD+**
**Variable**	**M (SD)**	**M (SD)**	**M (SD)**	**M (SD)**
Acts of physical IPV perpetration pretreatment	6.91 (6.61)	18.75 (34.87)	12.12 (17.41)	9.91 (20.65)
Acts of physical IPV perpetration posttreatment	0.82 (1.40)	0.38 (0.74)	7.84 (14.23)	3.17 (4.78)
Acts of verbal IPV perpetration pretreatment	24.27 (18.15)	47.75 (33.49)	37.00 (31.09)	29.41 (26.55)
Acts of verbal IPV perpetration posttreatment	12.27 (13.85)	8.62 (6.36)	34.67 (38.68)	14.71 (14.03)
Inflicted injuries pretreatment	2.91 (5.63)	1.75 (1.98)	1.32 (1.91)	2.54 (4.05)
Inflicted injuries posttreatment	0.09 (0.30)	0.00 (0.00)	1.00 (1.80)	1.21 (3.37)

IPV = intimate partner violence; I-StoP = Integrated treatment for Substance abuse and Partner violence; CBT-SUD+ = substance abuse treatment.

In both treatment conditions, the completers had committed significantly less physical IPV at posttreatment than at pretreatment (I-StoP: Z (10) = −2.68; p = .00 (1-tailed); CBT-SUD+: Z (7) = −2.37; p = .01 (1-tailed)). There were no significant differences between both conditions regarding posttreatment physical IPV perpetration after controlling for pretreatment physical IPV. ITT analyses yielded similar results. I-StoP patients and CBT-SUD+ patients both had committed significantly less acts of physical IPV at posttreatment (i.e., LOCF for dropouts) than at pretreatment (I-StoP: Z = −2.32; p = .01 (1-tailed); CBT-SUD+: Z = −2.87; p = .00 (1-tailed)). There were no differences between the treatments after controlling for pretreatment physical IPV perpetration.

Further, both treatments were effective in reducing verbal IPV perpetration among the completers (I-StoP: t (10) = 1.99; p = .04 (1-tailed); CBT-SUD+: t (7) = 3.31; p = .01 (1-tailed)), with no differences between the treatments after controlling for pretreatment verbal IPV perpetration. ITT analyses demonstrated that patients receiving I-StoP did not commit significantly less verbal IPV at posttreatment than at pretreatment (Z = −0.78; p = .19; 1-tailed), whereas participants receiving CBT-SUD+ did (Z = −2.76; p = .00; 1-tailed). However, after controlling for pretreatment verbal IPV perpetration, no differences between both treatments remained.

Finally, there was a non-significant trend that completers in both treatments had less often injured their partner at posttreatment than at pretreatment (I-StoP: Z = −1.83; p = .06 (1-tailed); CBT-SUD+: Z = −1.89; p = .06 (1-tailed)); with no significant posttreatment difference between both groups. ITT analyses demonstrated that participants allocated to I-StoP had not injured their partner significantly less often at posttreatment than at pretreatment (Z = −1.11; p = .13; 1-tailed), whereas patients receiving CBT-SUD+ did show a significant decrease in injuries inflicted (Z = −2.38; p = .01; 1-tailed). However, after controlling for pretreatment inflicted injuries, no differences between treatments remained.

#### Secondary outcome measures

Secondary outcome measures (general psychopathology, marital satisfaction and treatment satisfaction) of completers and ITT sample are displayed in Table 5.

**Table 5:** Secondary outcome measures of completers and ITT in both treatment conditions

	**Completers**		**Intention-to-treat sample**	
	**I-StoP**	**CBT-SUD+**	**I-StoP**	**CBT-SUD+**
**Variable**	**M (SD)**	**M (SD)**	**M (SD)**	**M (SD)**
BSI score–pretreatment	55.40 (29.91)	55.75 (33.39)	55.96 (37.91)	56.33 (34.01)
BSI score–posttreatment	26.10 (20.78)	25.38 (24.51)	41.08 (33.53)	46.00 (35.04)
MMQ score^1^–pretreatment	29.88 (18.36)	26.29 (12.65)	24.09 (16.98)	21.32 (12.39)
MMQ score–posttreatment	24.75 (15.68)	20.43 (23.22)	22.95 (15.55)	19.86 (15.89)
Treatment satisfaction–rating^2^	8.38 (1.16)	8.19 (0.65)	-	-

^1^ = the first five participants did not complete the MMQ since the instrument was added to the assessment in a later phase of the study; ^2^ = in both conditions, 8 participants evaluated treatment; BSI = brief symptom inventory; MMQ = Maudsley marital questionnaire; I-StoP = Integrated treatment for Substance abuse and Partner violence; CBT-SUD+ = substance abuse treatment.

BSI scores reflecting psychopathology of participants in both conditions were significantly lower at posttreatment than at pretreatment for completers (I-StoP: t (9) = 2.92; p = .01 (1-sided); CBT-SUD+: t (7) = 3.97; p = .00)) as well as ITT (I-StoP: t (24) = 2.69; p = .01 (1-sided); CBT-SUD+: t (23) = 2.69; p = .01 (1-sided)). There were no differences between both conditions. MMQ scores, reflecting marital satisfaction, had not significantly decreased (lower MMQ scores are associated with higher marital satisfaction) within conditions, nor were there any differences between the conditions for completers as well as ITT analyses. Completers in both conditions reported high rates of treatment satisfaction (on average over 8 out of ten). There were no differences in treatment satisfaction between conditions.

## Discussion

This is the first study that compared individual integrated CBT addressing both substance abuse and partner violence (I-StoP) to a bona fide substance use treatment as usual (i.e., CBT addressing substance abuse). Primary aim of the present study was to compare the effectiveness of I-StoP to substance use treatment as usual (with one session addressing IPV added; CBT-SUD+) in reducing physical IPV among patients referred for substance abuse treatment who were involved in a pattern of IPV perpetration. Analyses were conducted both on completers only and on all included patients (ITT sample). As expected, both I-StoP and CBT-SUD+ were effective in reducing substance use and no significant differences between both conditions were found; completers and ITT analyses yielded similar results. Further, in accordance with expectations, both treatments led to a significant decrease in physical IPV perpetration from pretreatment to posttreatment. However, contrary to expectations, I-StoP had not been superior to CBT-SUD+ in decreasing IPV. Again, the results on decrease in IPV were similar in completers and ITT analyses. Regarding secondary outcome measures, completers and ITT analyses showed slightly different outcomes. Completers analyses revealed a significant decrease in verbal IPV and inflicted injuries and a significant improvement in general psychopathology, with no differences between both treatments. In contrast, ITT analyses demonstrated that I-StoP had not led to less verbal IPV at posttreatment compared to pretreatment, whereas CBT-SUD+ had. This difference between conditions was significant. In addition, there was a non-significant trend that CBT-SUD+ had been effective in decreasing inflicted injuries, whereas I-StoP had not. Further, even though marital satisfaction at pretreatment was close to the maritally distressed range [85], both treatments (completers and ITT) did not improve marital satisfaction. Finally, completers in both conditions reported high rates of treatment satisfaction.

Although I-StoP was not more effective in reducing IPV than CBT-SUD+, the results of the current study are promising. Patients who completed treatment hardly engaged in IPV perpetration in the 8 weeks before treatment completion (I-StoP: M = 0.82 and CBT-SUD+: M = 0.38 acts of physical IPV perpetration at posttreatment). Therefore, the decrease of IPV perpetration after treatment completion was not only statistically significant but also clinically relevant. Moreover, the current study demonstrated that, on case level, patients with cannabis and/or cocaine use disorders also decreased IPV perpetration after substance abuse treatment. Since CBT-SUD+ is easier to implement in substance abuse treatment centers than I-StoP, it may be concluded from the above that CBT-SUD+, a CBT treatment addressing substance use disorders with the inclusion of one session addressing IPV, is a sufficient treatment to treat IPV perpetration in patients in substance abuse treatment.

Despite the favorable outcome, the results should be interpreted considering several limitations of the study. The foremost shortcoming is the high dropout rate (61.2%), which resulted in a completers sample of only 19 participants. Even though high dropout is common for patients in substance abuse treatment (a review by [86] described that the majority of studies reported dropout rates over 50% in the first month of treatment) and IPV treatment (ranging from about 20 to 70% [87]), dropout rates in this study were in the top range. Presumably, patients in whom both substance abuse and IPV perpetration are present are even more difficult to retain in treatment than patients with either problem alone. Also, dropout rates are comparable to dropout rates in a similar (pilot) study comparing the effectiveness of I-StoP to CBT addressing IPV alone among substance abusing IPV perpetrators referred to outpatient forensic treatment after committing IPV [Kraanen, Scholing, Emmelkamp: The comparative effectiveness of a combined substance abuse–partner violence treatment to partner violence treatment alone among patients in forensic outpatient treatment: A pilot study, submitted]. We argue however that because this study was conducted in routine clinical care, these dropout rates accurately reflect clinical practice, and that the external validity of this study is probably high. Further, it is also noticeable that low numbers of dropouts completed questionnaires (49% completed assessment halfway treatment, 39% completed posttreatment assessment), even though it has been tried repeatedly to obtain data by mailing questionnaires to patients and phoning them in order to remind participants to complete the forms. Unfortunately, it is common for patients in substance abuse treatment that they are difficult to contact after dropout, probably because they often suffer from various psychosocial problems as well.

Further, due to high dropout rates, statistical power for the completers sample (but not the ITT sample) was low. As described in the Methods section, 21 participants per condition were needed to detect a large difference between groups, tested 1-sided against alpha = .05 for a power of .80. For the ITT analyses, this number of 21 participants per condition was met, but not for the completers sample. Therefore, it is possible that true differences among completers (within groups as well as between groups) were not demonstrated. However, the main finding of the study is that the frequency of IPV had dropped to about zero in both treatment conditions for the patients who finished the treatment, independent of pretreatment severity of IPV.

Also, dropouts and completers were compared and were found not to differ regarding pretreatment physical IPV perpetration, verbal IPV perpetration, inflicted injuries, days of abstinence, substance use Z-scores, and pretreatment Axis-I disorders, except for major depressive episode. Chi-square tests demonstrated that dropouts were more often diagnosed with major depressive episode (N = 9; 29.0%) than completers (N = 1; 5.0%) (X^2^ (1) = 4.45; p = .04). This is in accordance with previous research; several studies have demonstrated that depression is linked to early treatment attrition (e.g., [88-90]). However, the fact that almost no differences were found between dropouts and completers might also be attributable to low statistical power (i.e., at least 64 or 21 participants per condition were necessary to demonstrate (according to [84]) medium or large effect sizes of Cohen’s f = .25 and .40, respectively; [83]). For example, the pretreatment physical IPV scores of completers who received I-StoP (M = 6.91) were obviously lower than ITT allocated to I-StoP (M = 12.12). In addition, since there were fewer patients who reported at least 7 acts of physical IPV in the past year than we had expected, we were unable to reach the target of 100 participants within the period that patients were recruited. Yet, despite the relatively small number of completers, IPV decreased significantly in the completers as well as the ITT sample. Nonetheless, future research should focus on finding approaches, apart from using motivational interviewing techniques, to increase treatment adherence and include more participants. For example, by targeting depressive symptoms from the beginning of treatment, as suggested by [90]. Also, expectancies regarding treatment should be assessed at the beginning of and during the course of treatment to examine whether treatments correspond with patients’ expectations and whether incorrect expectancies may be a reason for dropout.

Related to the high dropout and low retention rates is the fact that no ‘state of the art’ analyses could be used to analyze the results, such as multiple imputation (MI). MI performs well with dropout rates of at most 50% but power decreases fast as dropout rates get higher [91]. Further, MI demands that data are missing at random (MAR) (e.g., [92-94]). Many dropouts could not be contacted, but we assumed that they had probably relapsed and thus were not missing at random (NMAR). Moreover, the variables to be imputed exceeded the number of participants and imputing scale scores would lead to increased standard errors [91]. Since the data were probably NMAR, multilevel analysis could not be performed as well [79].

Another point of discussion is that the results were based on self-report, which may have been influenced by patients' tendency to underreport IPV perpetration. Alternative methods would have been using police records and/or partners reports as outcome. However, police records to verify IPV have a similar problem since only a small proportion of IPV is reported to the police (e.g., [95,96]). On the other hand, partner reports are also subject to limitations. [97] for example even demonstrated that victims reported less IPV than perpetrators. We have two reasons to assume that the findings in this study are fairly reliable and valid. 1) We tried to obtain pre- and posttreatment measures of partner on IPV by administering the CTS2. At pretreatment, 40.4% of partners completed the CTS2; at posttreatment this was only 7.7% of the total sample. Using these questionnaires as primary outcome measure would result in even lower completion rates. However, when comparing CTS2 physical IPV outcomes of both partners, we found no significant differences between participant and partner reports (Z = −0.82; p = .41), with partners reporting less acts of physical IPV (M = 6.5) than participants (M = 9.0). The second is that not only treatment progress but also study inclusion was based on self-disclosure of IPV. It is probable that some patients were not included at all because they underreported IPV, but the group who was finally included reported at least enough IPV to meet inclusion criteria. Another limitation is that the treatments in general lasted significantly longer than intended (on average 33 instead of 16 weeks). Reasons for this delay were, for example, that therapist and patient could not schedule sessions during certainweeks, were on vacation, or because patients were doing so well that they, in agreement with their therapist, did not need to come to the institution every week and instead preferred to spread treatment sessions over a longer period. This last point possibly implies that a shorter treatment might also be effective in reducing both substance use and partner violence. Further, results of the present study appear promising compared to the results of the meta-analyses by [31] and [32] that demonstrated that IPV treatment alone was hardly effective in reducing IPV. However, the comparison between the current study and these meta-analyses is difficult; the meta-analyses included follow-up data (6 months or more) whereas the current study only reported posttreatment results. Moreover, the meta-analyses included only police and partner reports compared to self-report in the present study. Further, although lacking follow-up results are also a limitation of the study, we argue that posttreatment and follow-up results are two different research topics that each have merits of their own. Finally, due to the small completers sample, it was not possible to add covariates to the analyses and control, for example, for participants’ own substance use, partner substance use and IPV victimization since statistical power would be too low.

The limitations described above lead to the following suggestions for future research. In the first place, it would be interesting to study whether a shorter treat-ment (of, for example, 10 sessions) would be as effective in reducing IPV perpetration as the 16 sessions counting CBT-SUD+ treatment. Further, since CBT-SUD+ contains 1 session addressing IPV, a prospective study could compare CBT-SUD+ to substance abuse treatment without addressing IPV to investigate whether it is indeed necessary to address IPV to reduce IPV perpetration or that substance abuse treatment alone is sufficient. Further, patients receiving inpatient treatment were excluded from the study. Future research should investigate whether CBT-SUD+ is as effective in reducing IPV in inpatients with more severe substance use disorders than in outpatients. In addition, predictors of treatment success should be studied. For example, there is substantial evidence that three or four subtypes of IPV perpetrators could be distinguished, i.e., the family only perpetrator who is only violent towards the partner, the borderline / dysphoric IPV perpetrator, who is characterized by psychic distress and is sometimes violent outside the family, and the antisocial IPV perpetrator (sometimes divided in two subtypes with different severity) who commits most extrafamilial violence of the subtypes [98,99]. It would be relevant to assess whether subtypes (or other patient characteristics) predict treatment success and/or dropout rates. Also, it should be examined whether type of substance use disorder predicts treatment outcome, whereas previous research only demonstrated that successfully treating alcohol dependence led to reductions in IPV perpetration. Although individual patients with alcohol, cannabis and/or cocaine use disorders who were included in the present study had improved with regard to IPV perpetration at posttreatment, unfortunately, the number of participants in this study was too low to analyze patients with different substance use disorders separately. Finally, it should be studied whether I-StoP is effective in reducing IPV perpetration in substance abusing patients who are referred to IPV treatment.

Despite its limitations, the study has several strengths. In the first place, it was demonstrated among patients entering substance abuse treatment that both treatments were successful in reducing IPV perpetration. Completers in both conditions almost did not perpetrate any IPV 8 weeks before the end of treatment compared to (on average) about 12 acts of physical IPV in the 8 weeks before starting treatment. Moreover, results are directly applicable to clinical practice.

## Conclusions

Based on the outcome of the present study we advise to routinely screen for IPV perpetration at intake, for example by using the J-IPV. After screening positive, IPV should be further assessed with, for instance, the CTS2. If IPV perpetration is confirmed, patients should be allocated to CBT-SUD+, since CBT-SUD+ is easier to implement in a substance abuse treatment center and therapists only need a limited amount of additional training to carry out the protocol. Moreover, since results indicated that it is not necessary to refer IPV perpetrating patients in substance abuse treatment to IPV treatment, CBT-SUD+ is a cost- and time-effective approach and it saves patients the inconvenience from attending treatment at two separate institutions. However, since this is the posttreatment report and effects of I-StoP may be delayed, follow-up results should be awaited to draw more firm conclusions regarding the comparative effectiveness of I-StoP and CBT-SUD+ and effectiveness of both treatments on the long term.

## Competing interests

The authors declare that they have no competing interests.

## Authors’ contributions

FLK, EV, AS, and PMGE contributed to the design of the study and development of the treatment protocols. EV contributed to implementation of the study. PMGE and FLK supervised therapists. FLK drafted the manuscript and EV, AS, and PMGE edited and added to the manuscript. All authors read and approved the final manuscript.

## Pre-publication history

The pre-publication history for this paper can be accessed here:

http://www.biomedcentral.com/1471-244X/13/189/prepub
